# AI-Based Quantitative HRCT for In-Hospital Adverse Outcomes and Exploratory Assessment of Reinfection in COVID-19

**DOI:** 10.3390/diagnostics15243156

**Published:** 2025-12-11

**Authors:** Xin-Yi Feng, Fei-Yao Wang, Si-Yu Jiang, Li-Heng Wang, Xin-Yue Chen, Shi-Bo Tang, Fan Yang, Rui Li

**Affiliations:** 1 Department of Radiology, Affiliated Hospital of North Sichuan Medical College, Nanchong 637000, China; 2 Department of Infectious Disease, Affiliated Hospital of North Sichuan Medical College, Nanchong 637000, China; 3 College of Medical and Dental Sciences, University of Birmingham, Birmingham B15 2TT, UK; 4 CT Collaboration, Siemens-Heathineers, Chengdu 610016, China

**Keywords:** COVID-19, high-resolution computed tomography (HRCT), deep learning, quantitative imaging, adverse outcomes, reinfection

## Abstract

**Background/Objectives:** Quantitative computed tomography (CT) metrics are widely used to assess pulmonary involvement and to predict short-term severity in coronavirus disease 2019 (COVID-19). However, it remains unclear whether baseline artificial intelligence (AI)-based quantitative high-resolution computed tomography (HRCT) metrics of pneumonia burden provide incremental prognostic value for in-hospital composite adverse outcomes beyond routine clinical factors, or whether these imaging-derived markers carry any exploratory signal for long-term severe acute respiratory syndrome coronavirus 2 (SARS-CoV-2) reinfection among hospitalized patients. Most existing imaging studies have focused on diagnosis and acute-phase prognosis, leaving a specific knowledge gap regarding AI-based quantitative HRCT correlates of early deterioration and subsequent reinfection in this population. To evaluate whether combining deep learning-derived, quantitative, HRCT features and clinical factors improve prediction of in-hospital composite adverse events and to explore their association with long-term reinfection in patients with COVID-19 pneumonia. **Methods:** In this single-center retrospective study, we analyzed 236 reverse-transcription polymerase chain reaction (RT-PCR)-confirmed COVID-19 patients who underwent baseline HRCT. Median follow-up durations were 7.65 days for in-hospital outcomes and 611 days for long-term outcomes. A pre-trained, adaptive, artificial-intelligence-based, prototype model (Siemens Healthineers) was used for pneumonia analysis. Inflammatory lung lesions were automatically segmented, and multiple quantitative metrics were extracted, including opacity score, volume and percentage of opacities and high-attenuation opacities, and mean Hounsfield units (HU) of the total lung and opacity. Patients were stratified based on receiver operating characteristic (ROC)-derived optimal thresholds, and multivariable Cox regression was used to identify predictors of the composite adverse outcome (intensive care unit [ICU] admission or all-cause death) and SARS-CoV-2 reinfection, defined as a second RT-PCR-confirmed episode of COVID-19 occurring ≥90 days after initial infection. **Results:** The composite adverse outcome occurred in 38 of 236 patients (16.1%). Higher AI-derived opacity burden was significantly associated with poorer outcomes; for example, opacity score cut-off of 5.5 yielded an area under the ROC curve (AUC) of 0.71 (95% confidence interval [CI] 0.62–0.79), and similar performance was observed for the volume and percentage of opacities and high-attenuation opacities (AUCs up to 0.71; all *p* < 0.05). After adjustment for age and comorbidities, selected HRCT metrics—including opacity score, percentage of opacities, and mean HU of the total lung (cut-off −662.38 HU; AUC 0.64, 95% CI 0.54–0.74)—remained independently associated with adverse events. Individual predictors demonstrated modest discriminatory ability, with C-indices of 0.59 for age, 0.57 for chronic obstructive pulmonary disease (COPD), 0.62 for opacity score, 0.63 for percentage of opacities, and 0.63 for mean total-lung HU, whereas a combined model integrating clinical and imaging variables improved prediction performance (C-index = 0.68, 95% CI: 0.57–0.80). During long-term follow-up, RT-PCR–confirmed reinfection occurred in 18 of 193 patients (9.3%). Higher baseline CT-derived metrics—particularly opacity score and both volume and percentage of high-attenuation opacities (percentage cut-off = 4.94%, AUC 0.69, 95% CI 0.60–0.79)—showed exploratory associations with SARS-CoV-2 reinfection. However, this analysis was constrained by the very small number of events (*n* = 18) and wide confidence intervals, indicating substantial statistical uncertainty. In this context, individual predictors again showed only modest C-indices (e.g., 0.62 for procalcitonin [PCT], 0.66 for opacity score, 0.66 for the volume and 0.64 for the percentage of high-attenuation opacities), whereas the combined model achieved an apparent C-index of 0.73 (95% CI 0.64–0.83), suggesting moderate discrimination in this underpowered exploratory reinfection sample that requires confirmation in external cohorts. **Conclusions:** Fully automated, deep learning-derived, quantitative HRCT parameters provide useful prognostic information for early in-hospital deterioration beyond routine clinical factors and offer preliminary, hypothesis-generating insights into long-term reinfection risk. The reinfection-related findings, however, require external validation and should be interpreted with caution given the small number of events and limited precision. In both settings, combining AI-based imaging and clinical variables yields better risk stratification than either modality alone.

## 1. Introduction

Coronavirus disease 2019 (COVID-19), caused by severe acute respiratory syndrome coronavirus 2 (SARS-CoV-2), has transitioned into an endemic phase globally, with intermittent resurgences driven by immune-evasive variants and changing population immunity [1]. As reinfection has become increasingly common in the post-Omicron era, identifying hospitalized patients at risk for early clinical deterioration or recurrent infection remains an important clinical concern [1,2,3].

Reverse-transcription polymerase chain reaction (RT-PCR) is the diagnostic gold standard for SARS-CoV-2 infection, but its sensitivity varies with sampling technique and viral load. High-resolution computed tomography (HRCT) has played a complementary role by sensitively detecting pulmonary involvement, especially during early infection or when RT-PCR results are inconclusive [4]. Traditional clinical risk scores and visual HRCT assessments, however, depend heavily on expert interpretation, may suffer from inter-observer variability, and often provide only coarse, semiquantitative estimates of disease extent.

These limitations restrict reproducibility, scalability, and the ability to capture subtle heterogeneity in lung involvement that may be relevant for risk stratification. Moreover, most early imaging studies emphasized short-term diagnostic or severity endpoints, and did not rigorously evaluate the incremental prognostic value of imaging beyond established clinical predictors [5,6,7,8,9].

Beyond diagnosis, HRCT provides detailed information on the extent and distribution of parenchymal involvement. Semiquantitative scoring systems can summarize overall lung burden but are time-consuming and may not be readily deployable in high-volume clinical settings.

Recent studies have suggested that AI-assisted, deep learning (DL)–based CT analysis enables automated, standardized, and reproducible assessment of lung involvement and may improve risk stratification for in-hospital outcomes, while substantially reducing processing time (e.g., to only a few seconds per case) and inter-observer variability [10]. Quantitative CT metrics—such as opacity score, lesion volume, and high-attenuation burden—have been used to stratify disease severity, predict acute outcomes, and track longitudinal changes in COVID-19 [11,12,13], and several recent AI-based studies have begun to integrate such quantitative CT or DL-derived imaging biomarkers with clinical variables, reporting improved prognostic performance and more refined characterization of post-COVID sequelae in selected cohorts [12,13].

Building on these developments, we focused on a vendor-provided AI tool that is already integrated into routine CT post-processing workflows, which offers practical advantages for real-world deployment. Specifically, we used the Siemens CT Pneumonia Analysis Prototype, a multi-vendor deep learning–based system that provides fully automated opacity- and attenuation-based quantitative metrics that are directly interpretable and can be readily combined with routine clinical variables. However, these approaches have primarily focused on acute in-hospital outcomes or long-term structural fibrosis, and only rarely have they formally quantified the added prognostic value of imaging on top of clinical factors. Critically, none have examined whether fully automated quantitative HRCT metrics at baseline relate to the risk of SARS-CoV-2 reinfection.

Meanwhile, reinfection has emerged as a clinically relevant issue, particularly among individuals with comorbidities or impaired immunity. Although prior infection and vaccination confer partial protection, immune escape, waning humoral responses, and persistent tissue-level alterations may predispose to recurrent episodes [14,15,16,17]. Observational cohorts have described heterogeneous patterns of immune durability and variable susceptibility to reinfection, yet robust imaging-derived indicators of reinfection risk remain poorly defined. Recent work has documented persistent radiologic abnormalities and delayed resolution after COVID-19 in a subset of patients, as well as long-term functional and immunologic perturbations [15,16,17,18]. Nevertheless, to our knowledge, no studies have systematically evaluated whether baseline AI-based quantitative HRCT features are associated with subsequent RT-PCR–confirmed SARS-CoV-2 reinfection in hospitalized patients.

Persistent structural abnormalities on HRCT—such as residual opacities, interlobular septal thickening, and high-attenuation regions—have been linked to ongoing epithelial injury, impaired mucociliary clearance, and dysregulated local immune responses [17,18,19]. These post-COVID alterations may weaken pulmonary barrier function and create a lung environment more permissive to subsequent infections, providing a biologically plausible rationale for exploring whether quantitative HRCT metrics of pneumonia burden can serve as indirect markers of vulnerability relevant to reinfection risk. At the same time, such imaging findings are non-specific and cannot be assumed to causally determine reinfection, underscoring the need for cautious, hypothesis-generating interpretation of any observed associations.

Given these gaps, there is a need for studies that (i) rigorously quantify the incremental prognostic value of automated, AI-based quantitative HRCT metrics beyond routine clinical variables for in-hospital composite adverse outcomes, and (ii) explore whether these imaging-derived markers carry any exploratory signal for long-term reinfection risk in patients with COVID-19 pneumonia.

Therefore, this study had two specific objectives: first, to assess whether combining deep learning–derived quantitative HRCT features with clinical factors improves prediction of a clearly defined composite in-hospital adverse outcome (intensive care unit admission or all-cause death) compared with clinical variables alone; and second, to explore the association between baseline quantitative HRCT metrics and subsequent RT-PCR–confirmed SARS-CoV-2 reinfection (≥90 days after the index infection) in a hospitalized cohort.

We hypothesized that fully automated quantitative HRCT parameters, when considered alongside clinical characteristics, would provide modest but measurable incremental prognostic information for early in-hospital deterioration, and that selected imaging metrics might show exploratory, hypothesis-generating associations with long-term reinfection risk without implying causation.

## 2. Materials and Methods

### 2.1. Study Design and Population

This retrospective, observational study screened consecutive patients with reverse-transcription polymerase chain reaction (RT-PCR)-confirmed SARS-CoV-2 infection who underwent high-resolution computed tomography (HRCT) between 1 November 2022, and 31 January 2023. After applying predefined inclusion and exclusion criteria, 236 patients were enrolled. The inclusion criteria were (1) age ≥ 18 years; (2) SARS-CoV-2 infection confirmed by RT-PCR using pharyngeal swab samples; and (3) availability of diagnostic-quality HRCT imaging. The exclusion criteria were (1) history of significant underlying lung diseases that could confound CT findings (e.g., lung cancer, tuberculosis, pneumoconiosis); (2) history of extrapulmonary malignancies or systemic wasting conditions (e.g., cachexia, end-stage disease); (3) poor image quality; (4) contrast-enhanced chest CT (as the prototype was trained for non-contrast evaluation); and (5) absence of pneumonia based on CT findings.

### 2.2. Follow-Up

For each patient, HRCT imaging and clinical data were collected, together with documentation of any concomitant fungal or bacterial pulmonary infection during hospitalization. Peak laboratory values at admission, including white blood cell count (WBC), lymphocyte percentage (LYM%), interleukin-6 (IL-6), D-dimer, C-reactive protein (CRP), and erythrocyte sedimentation rate (ESR), were also recorded.

Adverse outcomes during hospitalization were defined as either transfer to the intensive care unit (ICU) or all-cause mortality, and this composite endpoint was pre-specified prior to statistical analysis. In this study, SARS-CoV-2 reinfection was defined as a second RT-PCR-confirmed episode of COVID-19 occurring ≥90 days after the initial infection [20,21]. The primary hypothesis of this study was that combining deep learning–derived quantitative HRCT metrics with clinical variables would provide incremental prognostic information for the composite in-hospital adverse outcome compared with clinical variables alone, whereas a secondary, exploratory hypothesis was that selected baseline HRCT metrics would show an association with long-term RT-PCR–confirmed SARS-CoV-2 reinfection.

**Figure 1:**
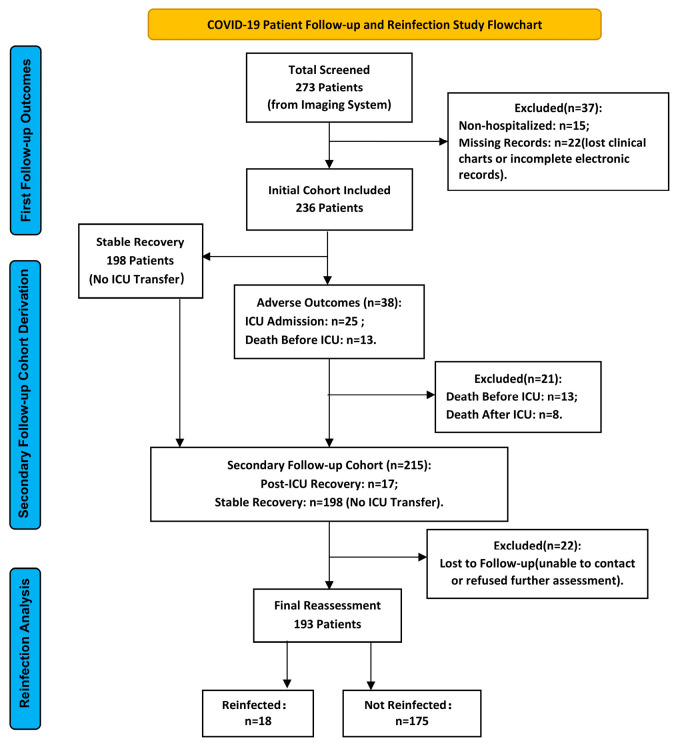
Flow chart showing the selection process for the study population. ICU, intensive care unit.

Follow-up data were obtained through a comprehensive review of serial electronic hospital records, including readmission logs and clinically indicated CT imaging reports. The observation period for adverse outcomes (ICU admission or all-cause mortality) began on the date of initial COVID-19 diagnosis and continued until the occurrence of an event or the last documented clinical encounter. For reinfection analysis, follow-up extended from hospital discharge (or, for non-hospitalized patients, the date of the last documented clinical or radiological assessment) until the earliest of the following: (1) laboratory-confirmed SARS-CoV-2 reinfection by RT-PCR; or (2) study termination (17 September 2024). The flow chart of the study population selection process is presented in Figure 1.

### 2.3. CT Examinations and Imaging Evaluation

All patients underwent chest HRCT on one of three scanners: Siemens SOMATOM Force (Siemens Healthineers, Erlangen, Germany), GE LightSpeed VCT (GE Healthcare, Milwaukee, WI, USA), or Philips Brilliance (Philips Healthcare, Best, The Netherlands). Scans were acquired with patients in the supine position during full inspiration, from the lung apices to the level of the adrenal glands. Raw data were reconstructed into thin-section images with a slice thickness and interval of 1.0–1.2 mm and transferred to a dedicated post-processing workstation for quantitative analysis using a deep learning-based pneumonia analysis prototype (Siemens Healthineers, Erlangen, Germany). The prototype operated offline on a local workstation without cloud-based data transmission, ensuring compliance with institutional data privacy and regulatory standards.

All scans were reconstructed using a soft-tissue convolution kernel, consistent with the requirements of the DL-based analysis prototype. Scanning protocols and reconstruction parameters were harmonized across the three CT platforms, and images were verified to meet routine clinical quality assurance standards before quantitative analysis. No explicit HU harmonization was applied across scanners; instead, we ensured protocol-level consistency and used a DL model validated for multi-vendor input.

To ensure accuracy, the initial segmentation results were independently reviewed by two radiologists (one with 13 and one with 4 years of CT diagnostic experience). All segmentations underwent visual quality control and were deemed acceptable for quantitative analysis. No manual corrections were applied, and all final quantitative outputs were based solely on the automated segmentation results generated by the prototype.

The Siemens CT Pneumonia Analysis Prototype, version 2.5.2 (Siemens Healthineers, Erlangen, Germany) used in this study is an offline, standalone AI-based tool designed for automated quantification of pneumonia-related abnormalities on non-contrast chest CT. Although not FDA-approved, it has undergone extensive multicenter validation.

Previous technical reports [22,23] describe that the prototype was trained on 1371 CT examinations for abnormality detection and on an additional dataset of 1000 COVID-19 CT scans, 131 interstitial lung disease cases, 113 bacterial pneumonia scans, and 559 normal CT examinations for lesion quantification. Its processing pipeline consists of multiple interpretable steps, including deep reinforcement learning–based anatomical landmark detection, DI2IN-based lung and lobe segmentation, and DenseUNet-based lesion segmentation using anisotropic convolutional kernels. In a multicenter evaluation involving 241 adult COVID-19 patients across the USA and Iran, only 2 CT scans (0.8%) required manual segmentation correction, and the tool achieved AUCs of 0.94–0.97 for severity prediction [23], indicating robust cross-scanner performance and high concordance with radiologist-verified segmentations.

In this study, it was used without retraining to ensure reproducibility across datasets. Consequently, no additional network training, fine-tuning, or convergence monitoring (e.g., training/validation loss curves) was performed as part of this study. The detailed layer-by-layer architecture (e.g., number of layers and filters per layer, kernel sizes, and total parameter count) and training hyperparameters (e.g., learning rate, batch size, and optimization schedule) of the DI2IN and DenseUNet modules are proprietary to the vendor and are not disclosed to end users; therefore, only high-level architectural information and training dataset composition, as reported in prior technical documentation [22,23], could be summarized in this manuscript, and the pretrained model was used as a fixed, unmodified tool. These characteristics made the prototype a pragmatic choice for our study, which aimed to evaluate the incremental prognostic value of AI-based quantitative HRCT in a real-world hospitalized COVID-19 cohort rather than to develop and train a new deep learning architecture.

**Figure 2:**
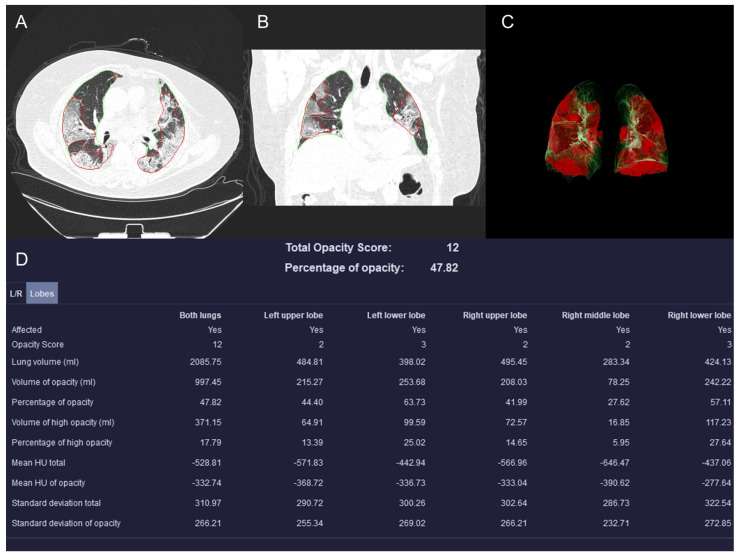
From a COVID-19 pneumonia patient: A 68-year-old male with comorbidities including diabetes mellitus, CHD, and COPD. During hospitalization, the patient developed a co-infection with acinetobacter baumannii. HRCT scans of the chest (diagrams (**A**–**C**)) revealed bilateral subpleural GGO. Diagram (**D**) demonstrates quantitative analysis results of various indicators using the prototype. The patient succumbed to the illness 4 days after admission. In (**A**–**C**), green denotes the segmented lung parenchyma and red indicates AI-identified pulmonary opacities (high-attenuation lesions).

The quantitative features extracted by the model include (1) total opacity score, calculated as the sum of five lobe scores, each graded on a scale from 0 to 4 based on the percentage of affected parenchyma: 0 = 0%, 1 = 1–25%, 2 = 26–50%, 3 = 51–75%, and 4 >75%; (2) lung volume (in milliliters) for the entire lung; (3) the volume and percentage of opacities; (4) the volume and percentage of high-attenuation opacities. The ≥−200 HU threshold used to define high-attenuation opacities is a fixed, built-in parameter of the Siemens AI prototype, reflecting the developer’s internal design for distinguishing denser consolidation from lower-attenuation ground-glass opacities; and (5) the mean HU value of the total lung and opacity. Representative segmentation and feature maps are illustrated in Figure 2.

In this study, all input DICOM series were processed using the CT Pneumonia Analysis Prototype with its default preprocessing pipeline. The software automatically converts the images to a standardized Hounsfield unit scale, restricts the analysis to the segmented lung fields, and filters out voxels outside the lung masks and small isolated noise components before feature extraction. We did not apply any additional in-house image preprocessing (e.g., manual smoothing, denoising, or resampling), and the deep learning model was used in inference-only mode without any retraining or data augmentation.

### 2.4. Statistical Analysis

All statistical analyses were performed using IBM SPSS Statistics (version 27.0; Armonk, NY, USA) and RStudio (Posit, version 2024.12.1 + 563; Boston, MA, USA). Continuous variables were tested for normality using the Shapiro–Wilk test and for homogeneity of variance using Levene’s test. Variables with a normal distribution were expressed as mean ± standard deviation (SD), whereas non-normally distributed variables were summarized as median and interquartile range (IQR; P25–P75).

Between-group comparisons were performed using the independent-samples *t* test or the Mann–Whitney U test for continuous variables, as appropriate, with Bonferroni correction applied for multiple comparisons involving HRCT parameters. Bonferroni correction was applied selectively and only to HRCT-derived quantitative parameters, which were numerous and correlated and thus more prone to inflated type I error under multiple testing.

In contrast, clinical and laboratory variables were fewer in number and served primarily as descriptive baseline assessments; applying Bonferroni correction to these variables would unnecessarily increase type II error. Categorical variables were reported as counts (percentages) and compared using the chi-square (χ^2^) or Fisher’s exact test, depending on expected cell frequencies.

To visually emphasize key between-group differences in selected clinical and imaging parameters, heatmaps were used to display normalized Z-scores, and boxplots were used to illustrate distributional differences. These visualizations were used to complement, rather than replace, the tabulated baseline comparisons. For visualization in heatmaps and boxplots, continuous clinical and quantitative HRCT variables were standardized to z-scores (mean = 0, standard deviation = 1) to allow comparison across different measurement scales; all hypothesis tests and regression models were fitted using the original (non-standardized) values or ROC-derived dichotomized variables. No additional data augmentation, synthetic sample generation, or class-rebalancing techniques were applied, and continuous variables were only screened for obvious data-entry errors based on clinical plausibility.

The predictive performance of clinical and imaging parameters for adverse outcomes was evaluated using receiver-operating-characteristic (ROC) curve analysis. Optimal cutoff values were derived from the Youden index and applied for risk stratification. The discriminative performance of individual predictors and multivariable models was summarized using the area under the ROC curve (AUC) and Harrell’s concordance index (C-index), which are standard metrics for time-to-event prognostic models. All ROC analyses and cutoff derivations were performed within the same dataset, without separate training or validation sets. Accordingly, the identified thresholds are exploratory and require external validation. Internal bootstrapping provides consistency estimates but does not constitute external validation. The cumulative incidence of adverse outcomes was estimated using Kaplan–Meier survival analysis, with between-group differences assessed by the log-rank test.

Univariate and multivariate Cox proportional-hazards regression models were used to identify independent risk factors for outcomes. The proportional hazards assumption was assessed using Schoenfeld residuals for each Cox model. Both global and individual covariate tests were performed to ensure validity of the model assumptions. Variables with *p* < 0.05 in univariate analysis or with established clinical relevance were entered into the multivariate model. Only variables with sufficient completeness and clear clinical interpretability were included to minimize model instability and avoid bias from extensive missingness.

Model performance was quantified by the concordance index (C-index), all statistical tests were two-sided, and a *p* value < 0.05 was considered statistically significant. Effect estimates (e.g., hazard ratios, odds ratios, AUCs, and C-indices) are reported with 95% confidence intervals (95% CIs) throughout. To assess internal model validity, bootstrap resampling was applied to compute optimism-corrected C-index values. Given the relatively small number of outcome events, we chose bootstrap internal validation rather than k-fold cross-validation, because bootstrap uses nearly the full dataset in each resample and is generally more efficient and stable than repeated data-splitting for Cox prognostic models. Model calibration was further assessed using bootstrapped calibration curves derived from Cox models for both outcomes.

For variables with missing values, mean imputation was applied to preserve sample size. Multiple imputation was considered; however, the proportion of missing data was minimal, and the affected variables were not included in the primary multivariable models. Given this low level of missingness, mean imputation was deemed a pragmatic, conservative approach for full-case analysis. Because lesion segmentation was fully automated and no manual intervention was applied, inter- and intra-observer reproducibility analyses were not required. To further assess the robustness of the Cox model results, multivariable logistic regression analyses were conducted. Imaging parameters identified as significant predictors in Cox regression were included in separate logistic models as binary variables.

**Figure 3:**
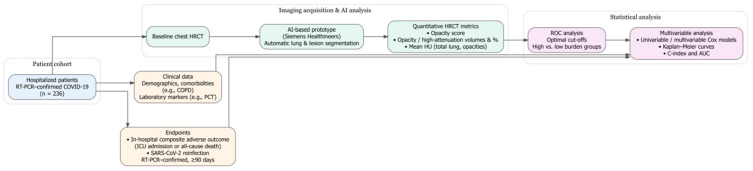
Workflow diagram of patient inclusion, AI-based quantitative HRCT analysis, endpoint definition, and statistical modeling.

A post hoc power analysis was conducted to evaluate whether the sample size was sufficient to detect clinically meaningful differences. Power estimates were calculated for both primary outcomes: in-hospital adverse events and long-term reinfection. The minimum required sample size was estimated based on effect sizes derived from multivariable Cox models, assuming 80% power and a two-sided α level of 0.05. To assess the robustness of the findings, a sensitivity analysis was performed by excluding one early death from the Cox regression model. An overview of the imaging analysis and statistical workflow is summarized in Figure 3.

## 3. Results

### 3.1. Patient Characteristics

A total of 273 patients who underwent HRCT during the COVID-19 epidemic period were initially screened from the imaging system. After excluding 15 non-hospitalized cases and 22 with missing clinical records, 236 patients with RT-PCR–confirmed COVID-19 pneumonia were included in the final analysis. The cohort was elderly and predominantly male (mean age 70.42 years, range 28–97 years; 148 men [62.7%]), and cardiometabolic comorbidities were common: approximately half of the patients had hypertension, and similar proportions had diabetes mellitus or coronary heart disease. More than 90% of patients had received at least one dose of COVID-19 vaccine at baseline. A total of 4.94 person-years (1804.4 person-days) of follow-up were accrued during the initial hospitalization period and 323.1 person-years (117,923 person-days) during the long-term reassessment period. During the initial hospitalization follow-up, the median observation period was 7.65 days (IQR: 5.23–11.36 days), and 38 patients (16.1%) experienced the composite in-hospital adverse outcome (ICU admission or all-cause death), whereas 198 patients (83.9%) did not, indicating a moderately imbalanced distribution of in-hospital events versus non-events.

For the long-term follow-up, 21 patients who died during the index hospitalization were excluded and 22 patients were lost to follow-up, leaving 193 survivors in the reinfection analysis cohort (median observation duration 611.00 days, IQR: 602.50–615.00 days). Among these patients, 18 (9.3%) were identified as having laboratory-confirmed SARS-CoV-2 reinfection, whereas 175 (90.7%) did not experience documented reinfection, resulting in a markedly imbalanced endpoint for the long-term analysis.

Compared with the non-adverse-outcome group, patients with adverse outcomes were significantly older and had higher levels of WBC, IL-6, D-dimer, CRP, and procalcitonin (PCT), along with lower lymphocyte percentage (LYM%) (all *p* < 0.05). Regarding quantitative imaging parameters, the adverse outcomes group demonstrated a higher opacity score, volume of opacities, percentage of opacities, volume of high-attenuation opacities, percentage of high-attenuation opacities, and mean HU of the total lung. The adverse-outcomes group also had a significantly higher prevalence of comorbidities, including cardiac insufficiency, coronary heart disease (CHD), COPD, and concurrent fungal or bacterial infections (*p* < 0.05; illustrated in Table 1).

Compared with the non-reinfection group, patients with reinfection showed no significant differences in age, sex distribution, smoking history, or major comorbidities such as hypertension, diabetes, coronary heart disease, or chronic obstructive pulmonary disease (all *p* > 0.05). Laboratory parameters—including WBC, lymphocyte percentage, IL-6, D-dimer, CRP, ESR, and PCT—were also comparable between the two groups (all *p* > 0.05), although a trend toward elevated PCT levels was observed in reinfected individuals (*p* = 0.02).

In contrast, quantitative HRCT parameters demonstrated notable differences. Reinfection cases exhibited significantly higher lung opacity scores (median = 10.50 vs. 6.00, *p* = 0.00), greater lesion volumes and percentages (median opacity volume = 829.10 mL vs. 365.03 mL, *p* = 0.01; percentage of opacities = 30.38% vs. 13.00%, *p* = 0.01), and a larger burden of high-attenuation opacities (volume = 204.79 mL vs. 55.46 mL, *p* = 0.00; percentage = 6.31% vs. 1.91%, *p* = 0.01). Baseline characteristics comparing patients with and without reinfection are presented in Table 2.

**Figure 4:**
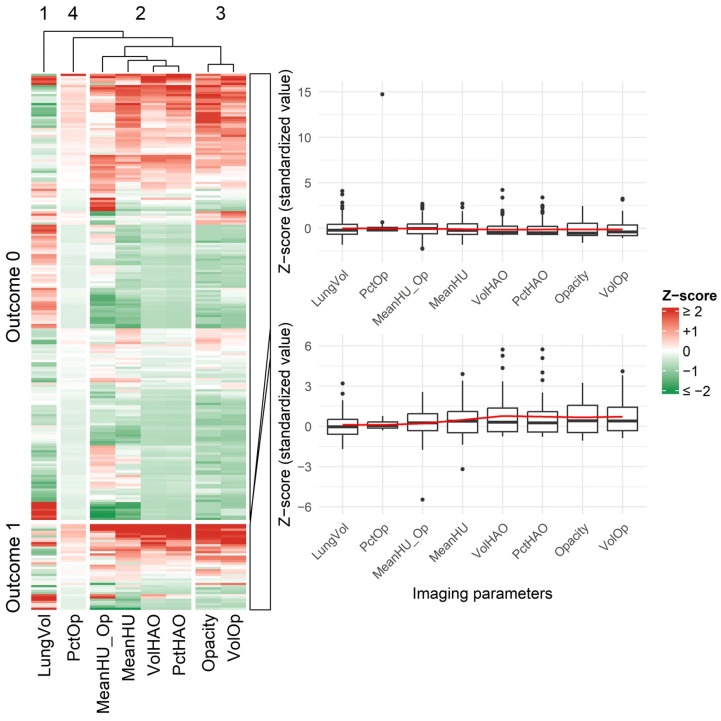
Heatmap and Boxplot of Baseline Chest CT Image Features in Patients With and Without Adverse Outcomes. Abbreviations: Opacity: Opacity score; LungVol: Lung volume; VolOp: Volume of opacities; PctOp: Percentage of opacities; VolHAO: Volume of high-attenuation opacities; PctHAO: Percentage of high-attenuation opacities; MeanHU: Mean Hounsfield Unit (total lung); MeanHU_Op: Mean HU of opacities. All imaging variables were standardized before plotting; heatmap colors and boxplots represent Z-scores (mean = 0, SD = 1).

**Figure 5:**
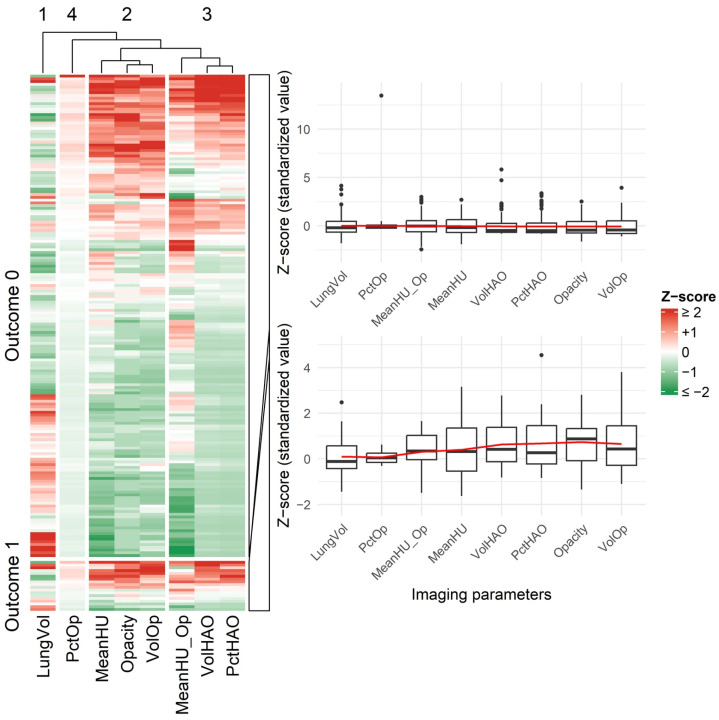
Heatmap and Boxplot of Baseline Chest CT Features in the Non-Reinfection and Reinfection Groups. Abbreviations: Opacity: Opacity score; LungVol: Lung volume; VolOp: Volume of opacities; PctOp: Percentage of opacities; VolHAO: Volume of high-attenuation opacities; PctHAO: Percentage of high-attenuation opacities; MeanHU: Mean Hounsfield Unit (entire lung); MeanHU_Op: Mean HU within opacities. All imaging variables were standardized before plotting; heatmap colors and boxplots represent Z-scores (mean = 0, SD = 1).

For both adverse-outcome and reinfection comparisons, Cohen’s d values were interpreted based on absolute magnitude: |0.2| = small, |0.5| = medium, and |0.8| = large effect sizes. Negative values reflect directionality of group differences rather than smaller effects. Across baseline characteristics, most clinical variables showed small effect sizes, whereas several HRCT-derived quantitative parameters—such as opacity score, volume of opacities, and high-attenuation metrics—demonstrated medium to large effects, indicating substantial group separation. Key between-group differences were also visualized to enhance interpretability: Figure 4 displays heatmaps and boxplots of baseline clinical and quantitative imaging features for adverse outcomes, and Figure 5 shows analogous comparisons by reinfection status.

### 3.2. Association Between HRCT Parameters and Adverse Outcomes

**Figure 6:**
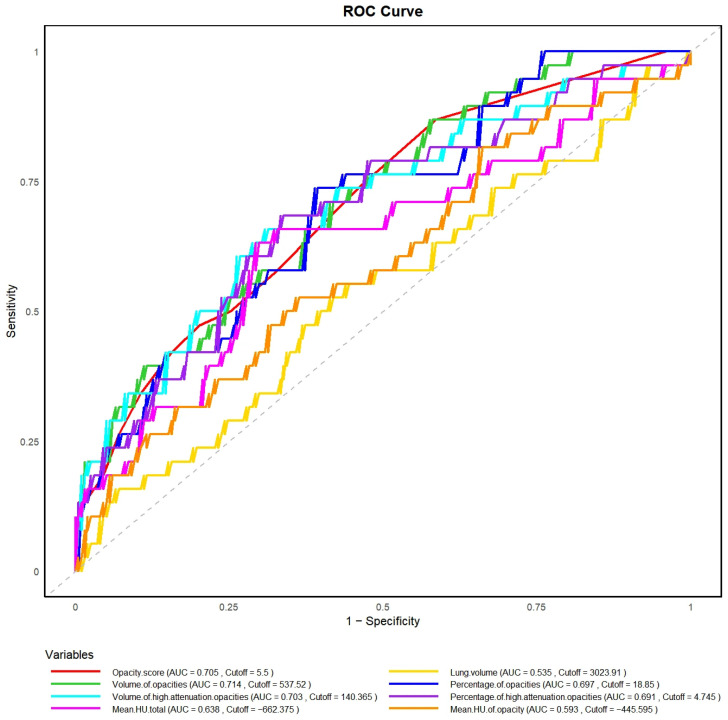
ROC Curve Analysis of Imaging Parameters in Predicting Adverse Outcome.

The predictive performance of imaging parameters was assessed using ROC curve analysis, with corresponding AUCs, optimal cutoff values, and ROC curves presented in Figure 6. Higher opacity scores (cut-off = 5.5, AUC 0.71, 95% CI 0.62–0.79), volume of opacities (cut-off = 537.52 mL, AUC 0.71, 95% CI 0.63–0.80), percentage of opacities (cut-off = 18.85%, AUC 0.70, 95% CI 0.61–0.79), and volume of high-attenuation opacities (cut-off = 140.37 mL, AUC 0.70, 95% CI 0.61–0.80) were significantly associated with poorer outcomes. Lung volume showed limited discrimination (cut-off = 3023.91 mL, AUC 0.54, 95% CI 0.43–0.64). Mean HU of the total lung (cut-off = −662.38 HU, AUC 0.64, 95% CI 0.54–0.74) and mean HU of opacities (cut-off = −445.60 HU, AUC 0.59, 95% CI 0.49–0.69) showed modest discrimination.

**Figure 7:**
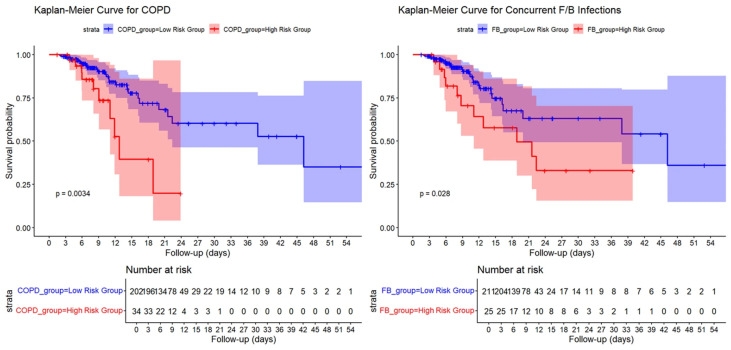
Kaplan–Meier Survival Curves by COPD and Concurrent Fungal/Bacterial Infection Status in COVID-19 Patients.

**Figure 8:**
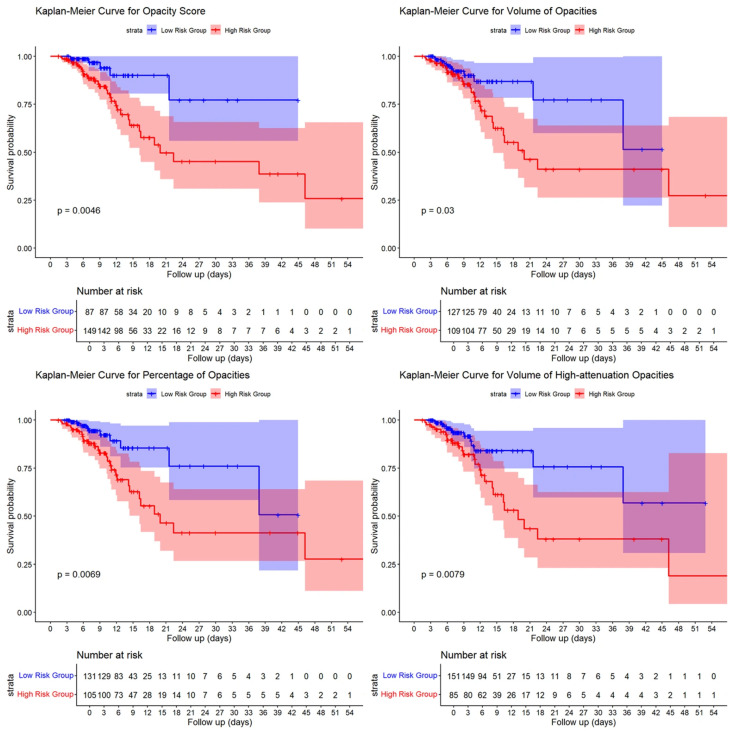
Kaplan–Meier Survival Curves by Opacity Score, Volume of Opacities, Percentage of Opacities and Volume of High-attenuation Opacities Stratification in COVID-19 Patients.

Kaplan–Meier survival curves were generated to evaluate the cumulative incidence of adverse outcomes during the follow-up period. Patients with higher opacity scores, greater volumes and percentages of pulmonary opacities, and larger volumes of high-attenuation opacities, as well as those with COPD or concurrent fungal/bacterial infections, exhibited significantly higher cumulative incidence rates of adverse outcomes and poorer prognoses (log-rank test, *p* < 0.05; see Figure 7 and Figure 8).

Univariate Cox regression analysis indicated that age, COPD, concurrent bacterial or fungal infections, IL-6, D-dimer, CRP, and high-value groups for opacity score, volume of opacities, percentage of opacities, volume of high-attenuation opacities and mean HU of the total lung were significantly associated with adverse outcomes (*p* < 0.05).

Multivariable Cox regression analysis demonstrated that opacity score ≥ 5.50 (adjusted HR = 3.02, 95% CI: 1.17–7.81, *p* = 0.02), percentage of opacities ≥ 18.85 (adjusted HR = 2.33, 95% CI: 1.12–4.84, *p* = 0.02), and mean HU of the total lung ≥ −662.38 (adjusted HR = 2.20, 95% CI: 1.12–4.30, *p* = 0.02) were independent predictors of adverse outcomes after adjusting for age and COPD. Because multivariable analysis of clinical factors identified age and COPD as independent predictors, subsequent Cox models evaluating imaging parameters were specifically adjusted for these two covariates (Table 3).

**Figure 9:**
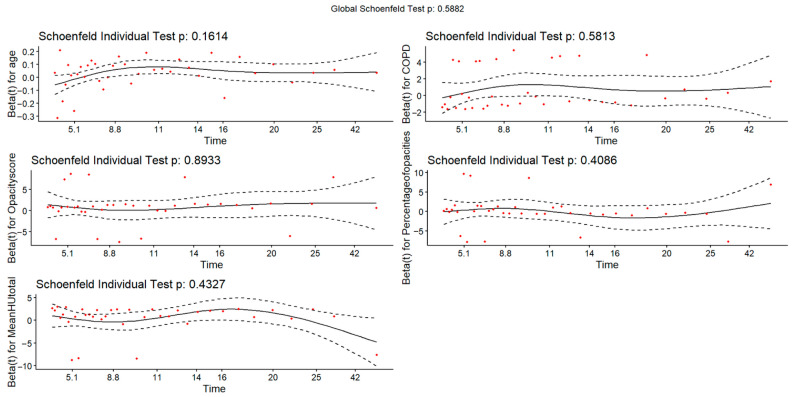
Schoenfeld Residuals for Proportional Hazards Assumption in Cox Regression Model of Adverse Outcomes. Red dots represent the scaled Schoenfeld residuals, the solid line is the fitted smoothed curve, and the dashed lines indicate the 95% confidence bands.

Assuming an event rate of 16% in the lower-risk group and 41% in the high-risk group, a minimum of 98 patients (49 per group) would be required to achieve 80% power. Our study included 236 patients in this analysis, indicating that the sample size was adequate to detect clinically meaningful differences. The proportional hazards assumption was tested using Schoenfeld residuals. No evidence of violation was observed for any covariate, and the global test was non-significant (*p* = 0.59). Residual plots showed no systematic trends over time. (see Figure 9).

To evaluate the robustness of the results, a sensitivity analysis was conducted by excluding one patient who died within 1.48 days of admission. Multivariate Cox regression models were re-estimated, and all key imaging predictors remained statistically significant. Specifically, an opacity score ≥ 5.50 was associated with an adjusted HR of 3.02 (95% CI: 1.07–8.54, *p* = 0.037); a percentage of opacities ≥ 18.85 had an HR of 2.33 (95% CI: 1.12–4.84, *p* = 0.023); and a mean HU of the total lung ≥ −662.38 yielded an HR of 2.20 (95% CI: 1.10–4.42, *p* = 0.025). These findings confirm that the observed associations were not driven by early mortality and remain robust after excluding this outlier case. Because multivariable logistic regression analyses yielded findings that were directionally consistent with the Cox models and did not provide additional prognostic insights, detailed logistic regression results are provided in the Supplementary Table S1 for completeness.

The C-index for age, COPD, opacity score, percentage of opacities, and mean HU of the total lung was 0.59 (95% CI: 0.46–0.71), 0.57 (95% CI: 0.49–0.65), 0.62 (95% CI: 0.55–0.69), 0.63 (95% CI: 0.54–0.72), 0.626 (95% CI: 0.53–0.72), respectively. The combined Cox regression model achieved a C-index of 0.68 (95% CI: 0.57–0.80), indicating modest discriminatory ability but an improvement over the individual predictors. Bootstrapping with 1000 resamples yielded a bias-corrected C-index of 0.640, suggesting small optimism (0.041) and supporting modest yet enhanced predictive performance when quantitative HRCT parameters are integrated with clinical variables.

**Figure 10:**
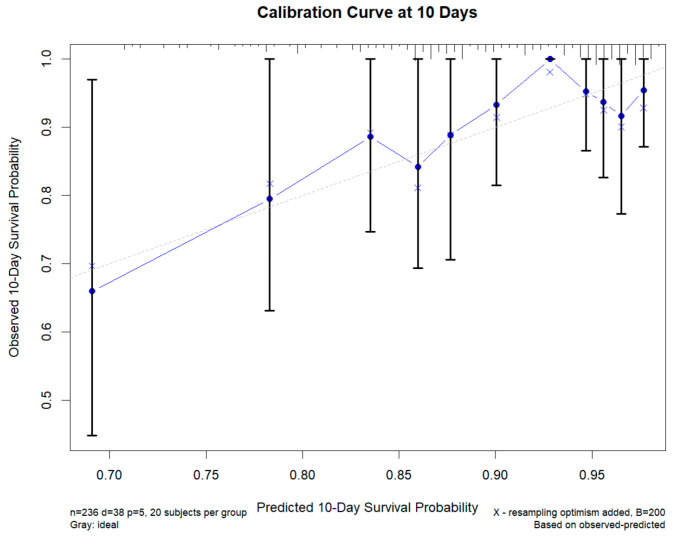
Calibration Curve of the Prediction Model for 10-Day Survival Probability. The grey dashed line represents the ideal reference line (perfect calibration), the blue connected points show the bias-corrected calibration curve, the ‘X’ symbols represent the optimism-corrected estimates from bootstrap resampling, and the black vertical bars indicate the 95% confidence intervals.

A calibration curve for the multivariable Cox model at 10 days (Figure 10) showed good agreement between predicted and observed outcomes. The 10-day calibration intercept was 0.60 (ideal = 0) and the calibration slope was 1.10 (ideal = 1), indicating only mild deviation from perfect calibration.

### 3.3. Association Between HRCT Parameters and SARS-CoV-2 Reinfection

During the long-term follow-up for reinfection assessment, univariate Cox regression analysis identified procalcitonin (PCT) and high-value groups for opacity score, volume of high-attenuation opacities, and percentage of high-attenuation opacities as significant predictors of SARS-CoV-2 reinfection (all *p* < 0.05).

After adjustment for PCT, multivariable analysis demonstrated that opacity score ≥ 5.50 (adjusted HR = 5.32, 95% CI: 1.21–23.46, *p* = 0.03), volume of high-attenuation opacities ≥ 140.37 mL (adjusted HR = 3.81, 95% CI: 1.46–9.97, *p* = 0.01), and percentage of high-attenuation opacities ≥ 4.935 (adjusted HR = 3.39, 95% CI: 1.29–8.90, *p* = 0.01) remained statistically associated with reinfection risk in this cohort, but the very wide confidence intervals for opacity score indicate substantial statistical uncertainty. Accordingly, these findings are considered exploratory rather than definitive (Table 3).

**Figure 11:**
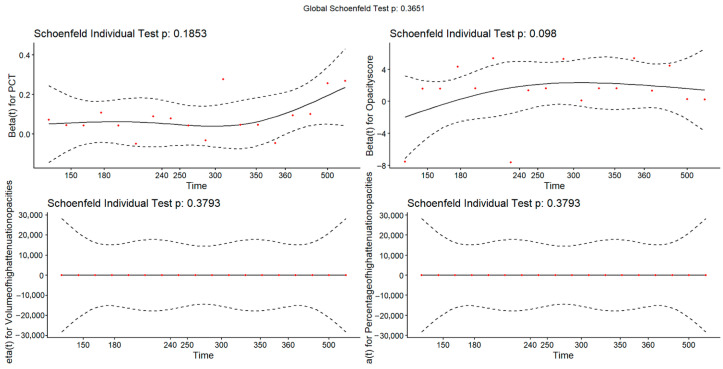
Schoenfeld Residuals for Proportional Hazards Assumption in Cox Regression Model of Reinfection. Red dots represent the scaled Schoenfeld residuals, the solid line is the fitted smoothed curve, and the dashed lines indicate the 95% confidence bands.

Given a baseline event rate of 9.3% and an estimated 40% in the high-risk group, a sample of 54 patients (27 per group) would be required to achieve 80% power. Our reinfection cohort included 193 patients, indicating that the estimated sample size was mathematically adequate, although the very wide CIs still suggest limited statistical stability. The proportional hazards assumption was evaluated using Schoenfeld residuals. All individual covariates met the assumption (all *p* > 0.05), and the global test was non-significant (*p* = 0.365). Residual plots also indicated no time-related trends. (see Figure 11). Because the logistic regression analyses for reinfection yielded results that were directionally consistent with the Cox models and did not provide additional prognostic insights, the detailed logistic regression outputs are presented in Supplementary Table S1.

In individual analyses, the discriminatory ability of single predictors was modest, with C-indices as follows: PCT (0.62, 95% CI: 0.47–0.78), opacity score (0.66, 95% CI: 0.58–0.74), volume of high-attenuation opacities (0.66, 95% CI: 0.54–0.77), and percentage of high-attenuation opacities (0.64, 95% CI: 0.53–0.76). In contrast, the combined model achieved a C-index of 0.73 (95% CI: 0.64–0.83), suggesting only moderate apparent discrimination within this dataset.

**Figure 12:**
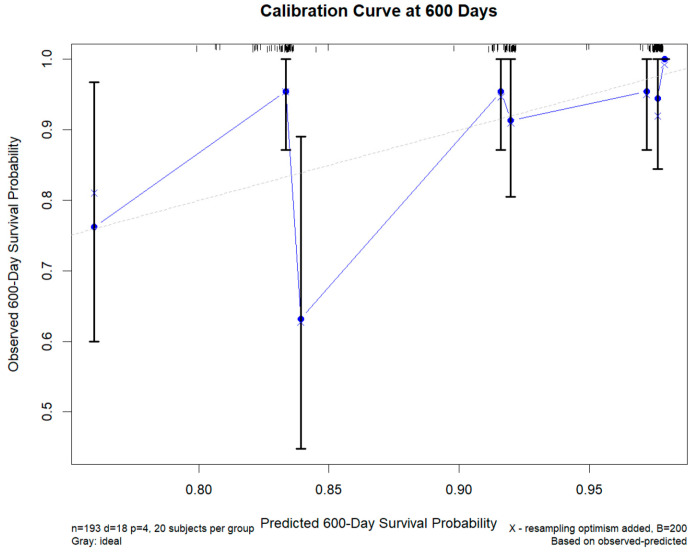
Calibration Curve of the Prediction Model for 600-Day Survival Probability. The grey dashed line represents the ideal reference line (perfect calibration), the blue connected points show the bias-corrected calibration curve, the ‘X’ symbols represent the optimism-corrected estimates from bootstrap resampling, and the black vertical bars indicate the 95% confidence intervals.

To internally validate this model, bootstrap resampling was performed. Due to occasional convergence issues, 954 successful iterations were completed. Bias-corrected estimates yielded internally consistent but still exploratory discrimination metrics. Calibration at 600 days (Figure 12) showed overall agreement between predicted and observed survival probabilities; however, the calibration intercept was 1.00 and the calibration slope was not estimable (slope = NA), reflecting quasi-complete separation arising from the small number of reinfection events and the use of dichotomized imaging predictors. These observations further underscore the exploratory nature of the reinfection model and indicate that its quantitative performance metrics should be interpreted with caution.

## 4. Discussion

This study identified both clinical and radiologic predictors of in-hospital adverse outcomes and explored potential imaging correlates of reinfection in patients with COVID-19 in northeastern Sichuan. In practical terms, these findings suggest that AI-derived opacity burden should be viewed as a complementary risk indicator rather than a stand-alone triage tool. A C-index of approximately 0.68 for the combined clinical–imaging model indicates moderate discrimination that is comparable to other imaging-based COVID-19 scores. In practice, this level of performance is most useful for flagging patients who may benefit from earlier escalation of monitoring and individualized supportive care, rather than for guiding definitive decisions on its own. We found that adverse outcomes were independently associated with age, COPD, and several quantitative HRCT parameters, whereas reinfection risk showed only exploratory associations with pneumonia-related imaging metrics in an underpowered analysis that should be interpreted cautiously. From a mechanistic standpoint, a higher baseline opacity or high-attenuation burden may reflect persistent epithelial injury, impaired mucociliary clearance, and dysregulated local immune responses, potentially increasing susceptibility to subsequent infections [15,24,25]. Nevertheless, alternative explanations, including detection bias, differences in health-care seeking, and unmeasured confounding (e.g., vaccination timing, antiviral use, or circulating variants), are also likely and may partly account for the observed associations [2,14]. Compared with prior studies, our findings further reinforce the prognostic value of HRCT-derived biomarkers—particularly opacity burden and high-attenuation volume—for stratifying risk beyond traditional clinical factors.

Previous studies have suggested that extensive lung involvement in COVID-19 may be associated with delayed viral clearance and immune dysregulation, which could contribute to prolonged disease course or risk of subsequent infection [26,27]. Our findings highlight the added value of combining imaging and clinical data for adverse-outcome prediction, while emphasizing that the reinfection-related results remain hypothesis-generating.

### 4.1. Clinical Implications

Age and COPD emerged as key predictors of adverse outcomes, which is consistent with prior studies showing that older adults have heightened endothelial dysfunction and prothrombotic risk [28] and COPD patients face greater vulnerability due to reduced pulmonary reserve and increased ACE2 expression [29]. These findings underscore the need for closer monitoring and tailored care in these populations.

Secondary infections also played a major role in poor prognosis. In our cohort, 29% of patients with adverse outcomes had concurrent fungal or bacterial infections, compared to 7% in those without these events. Immunosuppressive treatments like corticosteroids and tocilizumab, while beneficial for hyperinflammation, may predispose patients to co-infection [30,31]. These observations emphasize the importance of early infection screening, judicious antimicrobial use, and individualized immune support.

PCT also showed clinical utility. Although typically low in viral infections, elevated PCT in our study was independently associated with reinfection, potentially indicating bacterial co-infection or immune dysregulation [30,32,33]. As a marker that guides antibiotic decisions and improves outcomes in respiratory illness, PCT remains a valuable tool in COVID-19 management.

### 4.2. Radiologic Progression and Clinical Relevance

The prognostic value of HRCT in COVID-19 is well supported, and our quantitative results extend this evidence by leveraging AI-enhanced metrics. Pulmonary CT findings evolve dynamically over the disease course [5,34,35]. Early disease often presents with ground-glass opacities (GGOs), reflecting the exudative phase of diffuse alveolar damage (DAD), characterized by alveolar wall edema, inflammatory exudation, and hyaline membranes. Wider GGO distribution correlates with worse outcomes [36].

As disease progresses, consolidations and the “crazy-paving pattern” emerge—radiologically defined by GGOs with thickened inter- and intra-lobular septa—and correspond histopathologically to mixed-phase DAD with intra-alveolar fibrin and early fibrosis (fibroblast proliferation, collagen deposition, septal thickening) [37,38]. Late-stage features include linear/reticular opacities, fibrous stripes, subpleural lines, and fibrotic changes; GGOs may persist or extend but often decrease in density with re-expansion. Subsegmental atelectasis can distort bronchovascular bundles [39]. Fibrosis-like changes (e.g., bronchiectasis, reticulation) are more frequent at discharge in severe cases and may imply lasting functional impairment [40]. HRCT is particularly valuable for severity grading and longitudinal assessment, as dynamic changes in parenchymal involvement reflect disease progression and correlate with clinical outcomes [9].

### 4.3. Quantitative Imaging and AI Integration

Beyond qualitative interpretation, AI-based quantitative CT provides objective surrogates of parenchymal disease burden and has demonstrated substantial promise in predicting clinical outcomes in COVID-19. Previous studies have shown that reduced, well-aerated lung volume in ARDS correlates with disease severity [41], and semi-quantitative assessments of affected lung volume can accurately predict oxygen demand and the need for intubation [6].

In our study, a deep learning-based prototype automatically extracted key imaging biomarkers from HRCT scans, including opacity score and high-attenuation lesion metrics. ROC-derived thresholds allowed dichotomization into clinically meaningful risk groups. These opacity-derived indices demonstrated strong and independent associations with adverse outcomes and only exploratory, statistically unstable associations with SARS-CoV-2 reinfection, reflecting limited power in the reinfection analysis. Prior research using the same AI prototype has shown that combining total opacity volume with the percentage of high-attenuation opacities outperforms subjective severity scoring in predicting clinical deterioration [23].

Our results are consistent with external validations. For instance, Chabi et al. reported that in a cohort of 323 hospitalized COVID-19 patients, AI-quantified total opacity and consolidation volumes independently predicted death or ICU admission and improved model performance when combined with clinical variables (AUC = 0.83 vs. 0.76 for clinical data alone) [42]. Similarly, Grodecki et al. compared AI-based CT quantification with visual scoring in 743 patients, showing higher predictive accuracy for deterioration or mortality (AUC = 0.739 vs. 0.711) and an approximately 80% reduction in processing time [10]. Pang et al. demonstrated that the percentage of pneumonia volume (PPV) at admission predicted progression to critical illness (AUC = 0.868), outperforming traditional inflammatory biomarkers [43]. Together, these studies support the utility of AI-derived opacity and attenuation metrics as robust, reproducible predictors of clinical deterioration, often outperforming subjective visual assessments.

In our cohort, higher mean total-lung attenuation at baseline was associated with worse clinical outcomes, likely reflecting a radiologic transition from GGOs to dense consolidations. Such consolidations are pathophysiologically linked to alveolar collapse and microcirculatory impairment, contributing to ventilation–perfusion mismatch and progressive respiratory failure [8]. Although these findings are biologically plausible, prospective validation of attenuation-based thresholds is needed before they can be incorporated into routine clinical decision-making.

To account for anatomical variation, opacity volumes were normalized to total lung volume, yielding both an opacity percentage and a composite opacity score—both of which were significant prognostic markers. These findings are consistent with prior studies reporting higher opacity burden in severe COVID-19 [44,45,46]. While quantitative approaches improve objectivity and reproducibility, inter-institutional harmonization of imaging protocols and AI models remains essential for broad generalization.

Given the very small number of reinfection events and the wide confidence intervals of reinfection-related HRs, the observed association between high-attenuation lesions and reinfection risk should be interpreted as hypothesis-generating rather than causal. While our findings suggest an association between high-attenuation lesions and reinfection, previous work indicates that persistent parenchymal injury and abnormal repair may contribute to impaired pulmonary defense mechanisms, thereby warranting further mechanistic validation [24,25,45]. These imaging findings may reflect a pulmonary environment more susceptible to subsequent infections, but further mechanistic and prospective studies are necessary to confirm this relationship. If validated in prospective cohorts, such imaging biomarkers might help to inform follow-up strategies and long-term management in selected high-risk populations.

Comparative studies have demonstrated that AI-based quantitative CT analysis surpasses traditional visual CT severity scores in predictive accuracy and reproducibility, often achieving higher AUCs and markedly reducing processing time. Moreover, radiomic-enhanced models integrating quantitative metrics further improve prognostic discrimination. Nonetheless, inter-software variability and the need for harmonized AI pipelines remain critical barriers to universal clinical adoption [7,10,47].

The C-indices for our models ranged from 0.59 to 0.73, indicating modest discriminative ability. A value of 0.5 represents no better than random prediction, while values exceeding 0.7 are generally considered acceptable in clinical prognostic models. Despite this moderate performance, our models demonstrated reasonable predictive utility in a real-world, heterogeneous clinical population. Given the limited number of reinfection events, these results should be interpreted with caution and considered hypothesis-generating, warranting further validation through mechanistic studies and prospective longitudinal cohorts.

Although deep learning systems are often criticized for their “black-box” nature, the Siemens CT Pneumonia Analysis Prototype used in this study is based on a transparent and anatomically constrained segmentation pipeline. The system incorporates well-defined modules—including deep-reinforcement-learning–based landmark detection, DI2IN-based lung and lobe segmentation, and DenseUNet-based lesion segmentation—that allow for an interpretable, stepwise processing architecture. Importantly, the quantitative outputs generated by the prototype (such as opacity volume, percentage of affected lung, and high-attenuation opacity burden) are fully explainable radiologic metrics rather than latent or non-interpretable deep features. Furthermore, prior multicenter evaluations of the same prototype have demonstrated high concordance with radiologist-verified segmentations (only 0.8% of cases required manual correction) and stable performance across scanners from different vendors, supporting both the reliability and cross-scanner robustness of the model [22,23]. Taken together, these characteristics provide reassurance regarding the transparency of the analytical process and the clinical interpretability of the quantitative CT measures used in this study.

### 4.4. Novelty Highlights

This study offers three main novel contributions. First, it evaluates a fully automated, vendor-developed deep learning HRCT quantification tool in a hospitalized COVID-19 cohort and formally quantifies its incremental prognostic value beyond established clinical predictors for a clearly defined composite in-hospital adverse outcome. Second, it explores whether baseline opacity burden and high-attenuation metrics, summarized using ROC-derived thresholds, are associated not only with in-hospital deterioration but also with long-term RT-PCR–confirmed reinfection, thereby extending quantitative HRCT risk stratification to a longer time horizon. Third, it benchmarks clinical-only versus combined clinical–imaging models with internal bootstrap validation and calibration analyses, providing a pragmatic, workflow-compatible framework that can inform future scaling and deployment of AI-assisted quantitative CT in real-world practice and in future respiratory outbreaks.

## 5. Conclusions

In this single-center retrospective study, deep learning-derived quantitative HRCT metrics, combined with clinical variables, provided useful prognostic information for in-hospital adverse outcomes in patients with COVID-19 pneumonia. These fully automated quantitative HRCT metrics are derived from routine non-contrast chest CT scans and can be computed on standard post-processing workstations without additional radiation or acquisition time, supporting a feasible pathway for incorporating quantitative CT into early risk stratification and closer monitoring, particularly when critical-care resources are constrained. The same framework may be transferable to other viral or inflammatory pneumonias and future respiratory outbreaks, but will require multi-center prospective validation across different vendors and scanners, streamlined integration into PACS/CT reporting workflows, and formal evaluation of its impact on clinician decision-making and resource allocation in diverse health-care settings.

In contrast, the associations between baseline CT metrics and long-term SARS-CoV-2 reinfection were based on a very small number of events and yielded wide confidence intervals. These reinfection-related findings are therefore exploratory and hypothesis-generating, require external validation, and should not be used to guide clinical decision-making at this stage. At present, they are best regarded as tentative signals about possible structural correlates of post-COVID vulnerability, primarily useful for informing the design of future mechanistic and longitudinal studies rather than for direct bedside decisions. In the absence of immunologic assessments, vaccination timing, and viral variant data, these associations more likely represent indirect reflections of underlying pulmonary vulnerability rather than true determinants of reinfection risk.

### Limitations and Risk-Mitigation Strategies

This study has several limitations. Some biomarkers (e.g., IL-6) exhibit diurnal variation, and standardized cutoffs or sampling windows are lacking. The single-center design and modest sample size limit external validity. All patients were recruited from a single institution in southwestern China, which may not represent broader or international populations. Because this study included only hospitalized patients who underwent HRCT, outpatients with milder disease were not captured, which may introduce selection bias and limit generalizability.

Imaging data were processed using a proprietary Siemens AI prototype, which may not be generalizable across different scanner platforms, institutions, or clinical workflows. Importantly, no HU harmonization across scanners was performed. Although most examinations were obtained on similar Siemens systems, even minor differences in scanner calibration or reconstruction parameters could influence attenuation-based measurements (e.g., mean lung density, high-attenuation opacity volume), potentially introducing variability into these quantitative CT metrics.

Notably, we did not perform an independent validation or report quantitative performance metrics (e.g., Dice coefficient, validation accuracy) for the deep learning prototype used, as these were not available from the original developer. All AI-generated segmentation masks underwent visual quality control by two radiologists and were deemed acceptable for quantitative analysis. Because quantitative results relied entirely on automated masks without manual refinement, undetected segmentation inaccuracies—although none were identified on review—cannot be fully excluded.

In addition, although vaccination status was recorded, the number of doses and vaccine types administered were not systematically documented, which could have influenced reinfection risk and immune response. Furthermore, several clinically important covariates—including oxygen requirement, vital signs, antiviral treatment status, vaccination timing, and viral variant information—were incompletely recorded in the retrospective dataset and therefore could not be incorporated into the multivariable models. The absence of these variables may have introduced residual confounding and affected both adverse-outcome and reinfection analyses.

To partially mitigate these limitations and reduce potential risks of bias and misinterpretation, we used a prespecified composite endpoint for in-hospital adverse outcomes, performed visual quality control of all AI-generated segmentation masks by two radiologists before quantitative analysis, and internally assessed model performance using bootstrap resampling and calibration analyses. Nevertheless, residual confounding, spectrum bias, and overfitting cannot be fully excluded—particularly for the underpowered reinfection analysis—and the reported performance metrics should therefore be interpreted with appropriate caution.

These factors collectively constrain the external generalizability of our findings. In particular, the reinfection analysis—based on only 18 events—is clearly underpowered, and the very wide confidence intervals for reinfection-related HRs indicate substantial statistical instability and an increased risk of overfitting. We also did not perform a formal ablation study to dissect the relative contribution of individual imaging features and clinical variables (e.g., clinical-only vs. imaging-only vs. combined models, or stepwise removal of specific HRCT metrics), nor did we perform k-fold cross-validation, because the limited number of outcome events (38 in-hospital adverse events and 18 reinfections) would likely have produced unstable estimates. Although internal bootstrapping was performed, this cannot fully overcome optimism bias or substitute for external validation.

Future research should include prospective, multicenter validation studies using harmonized AI pipelines and standardized acquisition protocols to enhance reproducibility and generalizability. Comprehensive, systematically collected data on treatments, respiratory support requirements, immunization history, vital signs, and viral genotyping should also be incorporated to mitigate residual confounding factors and improve clinical relevance. Despite these constraints, ongoing advances in AI-driven imaging analysis may eventually support the development of more robust, personalized decision–support tools for future respiratory outbreaks, provided that rigorous external validation and careful assessment of clinical impact are performed.

## Figures and Tables

**Table 1:** Baseline Characteristics of Patients in the Adverse Outcomes Group and Non-Adverse Outcomes Group.

Parameter	Non-Adverse Outcomes Group (*n* = 198)	Adverse Outcomes Group (*n* = 38)	z/χ^2^	*p*	Cohen’s *d*
Clinical Characteristics					
Age, year	73.00 (59.00, 81.00)	78.00 (71.50, 84.25)	−2.66	0.01	−0.47
Male, *n* (%)	120 (60.61)	28 (73.68)	2.33	0.13	NA
Smoke, *n* (%)	35 (17.68)	9 (23.68)	0.76	0.38	NA
Hypertension, *n* (%)	96 (48.49)	17 (44.74)	0.18	0.67	NA
Diabetes, *n* (%)	94 (47.48)	23 (60.53)	2.17	0.14	NA
Cardiac insufficiency, *n* (%)	31 (15.66)	14 (36.84)	9.27	0.00	NA
CHD, *n* (%)	92 (46.47)	25 (65.79)	4.76	0.03	NA
Cerebral infarction	31 (15.66)	6 (15.79)	0	0.98	NA
Bronchiectasis, *n* (%)	11 (5.56)	1 (2.63)	0.57	0.45	NA
COPD, *n* (%)	24 (12.12)	10 (26.32)	5.21	0.02	NA
Emphysema, *n* (%)	48 (24.24)	13 (34.21)	1.65	0.20	NA
Asthma, *n* (%)	2 (1.01)	0 (0.00)	0.39	0.53	NA
Concurrent with F/B, *n* (%)	14 (7.07)	11 (28.95)	16.11	0.00	NA
Vaccinated, *n* (%)	182 (91.92)	35 (92.11)	0	1.00	NA
Laboratory Parameters					
WBC, ×10^9^/L	6.69 (5.23, 9.19)	9.63 (5.34, 13.57)	−2.42	0.02	−0.02
LYM%	14.30 (9.25, 20.60)	6.35 (3.60, 10.03)	−4.83	0.00	0.52
IL-6, ng/L	27.38 (8.13, 109.68)	109.68 (49.56, 274.00)	−5.37	0.00	−0.86
D-dimer, mg/L	1.77 (1.15, 4.78)	5.07 (2.18, 15.54)	−4.86	0.00	−1.20
CRP, mg/L	63.78 (15.68, 81.33)	84.61 (44.07, 154.70)	−3.48	0.00	−0.72
PCT, mg/mL	0.09 (0.05, 0.75)	0.78 (0.17, 1.46)	−4.61	0.00	−0.39
ESR, mm/h	58.92 (48.00, 60.50)	58.92 (42.75, 65.25)	−0.18	0.86	−0.03
CT-Based Lung Parameters					
Opacity score	6.00 (5.00, 10.00)	9.50 (6.00, 14.00)	−4.04	0.00	−0.84
Lung volume, mL	2936.33 (2523.18, 3541.56)	3107.06 (2555.71, 3670.27)	−0.69	0.49	−0.15
Volume of opacities, mL	426.30 (164.62, 906.45)	925.03 (452.09, 1571.74)	−4.18	0.00	−0.92
Percentage of opacities, %	13.33 (5.03, 34.33)	32.78 (14.53, 55.98)	−3.85	0.00	−0.13
Volume of high-attenuation opacities, mL	63.48 (22.50, 214.23)	236.28 (67.10, 469.82)	−3.97	0.00	−0.99
Percentage of high-attenuation opacities, %	2.04 (0.70, 7.31)	7.67 (2.53, 14.84)	−3.73	0.00	−0.90
Mean HU total, HU	−714.53 (−763.02, −639.27)	−649.66 (−742.91, −572.64)	−2.70	0.01	−0.58
Mean HU of opacity, HU	−471.04 (−530.67, −421.24)	−443.12 (−502.80, −367.68)	−1.81	0.07	−0.25

Data are presented as interquartile range and *n* (%), *n* (%) indicate the proportion of patients with the given condition in each group; percentages within a column do not sum to 100% because comorbidities are not mutually exclusive and the ‘No’ category is not shown. Abbreviations: CHD, coronary heart disease; COPD, chronic obstructive pulmonary disease; F/B, Fungal//Bacterial; WBC, white blood cell count; LYM%, lymphocyte percentage; IL-6, interleukin-6; CRP, C-reactive protein; PCT, procalcitonin; ESR, erythrocyte sedimentation rate. The Mann–Whitney U test was applied for continuous variables, and the Chi-square test or Fisher’s exact test was used for categorical variables, as appropriate.

**Table 2:** Baseline Characteristics of Patients in the Non-Reinfection Group and Reinfection Group.

Parameter	Non-Reinfection Group (*n* = 175)	Reinfection Group (*n* = 18)	z/χ^2^	*p*	Cohen’s *d*
Clinical Characteristics					
Age, year	72.00 (59.00, 81.00)	78.50 (63.50, 84.00)	−1.06	0.28	−0.18
Male, *n* (%)	105 (60.00)	12 (66.67)	0.30	0.58	NA
Smoke, *n* (%)	31 (17.71)	5 (27.78)	1.09	0.34	NA
Hypertension, *n* (%)	86 (49.14)	6 (33.33)	1.64	0.20	NA
Diabetes, *n* (%)	83 (47.43)	7 (38.89)	0.48	0.49	NA
Cardiac insufficiency, *n* (%)	28 (16.00)	2 (11.11)	0.30	0.74	NA
CHD, *n* (%)	80 (45.71)	9 (50.00)	0.12	0.73	NA
Cerebral infarction	25 (14.29)	4 (22.22)	0.81	0.48	NA
Bronchiectasis, *n* (%)	7 (4.00)	2 (11.11)	1.86	0.20	NA
COPD, *n* (%)	22 (12.57)	2 (11.11)	0.03	1.00	NA
Emphysema, *n* (%)	41 (23.429)	5 (27.78)	0.17	0.77	NA
Asthma, *n* (%)	2 (1.14)	0 (0.00)	0.21	1.00	NA
Concurrent with F/B, *n* (%)	12 (6.86)	2 (11.11)	0.44	0.63	NA
Vaccinated, *n* (%)	165 (94.29)	14 (93.33)	0.02	1.00	NA
Laboratory Parameters					
WBC, ×10^9^/L	6.69 (5.30, 9.19)	7.01 (4.66, 9.16)	−0.27	0.79	0.08
LYM%	14.40 (9.40, 21.30)	13.10 (7.95, 18.40)	−0.92	0.36	0.27
IL-6, ng/L	24.26 (7.33, 109.68)	58.23 (17.77, 109.68)	−1.34	0.18	−0.03
D-dimer, mg/L	1.75 (1.06, 5.01)	2.02 (1.36, 4.48)	−0.80	0.43	0.09
CRP, mg/L	62.75 (13.91, 81.70)	71.43 (49.81, 81.14)	−1.23	0.22	−0.34
PCT, mg/mL	0.08 (0.05, 0.59)	0.60 (0.05, 8.27)	−1.94	0.05	−0.99
ESR, mm/h	58.92 (48.00, 59.00)	58.92 (58.92, 74.00)	−1.43	0.15	−0.20
CT-Based Lung Parameters					
Opacity score	6.00 (5.00, 9.00)	10.50 (6.75, 12.25)	−3.12	0.00	−0.83
Lung volume, mL	2943.86 (2513.01, 3552.76)	3024.37 (2716.19, 3825.59)	−0.50	0.62	−0.11
Volume of opacities, mL	365.03 (165.00, 875.37)	829.10 (437.09, 1478.28)	−2.57	0.01	−0.73
Percentage of opacities, %	13.00 (4.71, 32.34)	30.38 (11.97, 51.28)	−2.47	0.01	−0.07
Volume of high-attenuation opacities, mL	55.46 (22.20, 182.03)	204.79 (105.81, 371.80)	−2.93	0.00	−0.71
Percentage of high-attenuation opacities, %	1.91 (0.68, 6.39)	6.31 (3.01, 13.15)	−2.63	0.01	−0.76
Mean HU total, HU	−715.37 (−764.83, −640.42)	−669.24 (−757.24, −560.53)	−1.44	0.15	−0.44
Mean HU of opacity, HU	−474.12 (−530.57, −423.33)	−440.94 (−491.70, −371.55)	−1.66	0.10	−0.34

Data are presented as median (interquartile range) for continuous variables and *n* (%) for categorical variables. Abbreviations: CHD, coronary heart disease; COPD, chronic obstructive pulmonary disease; F/B, fungal/bacterial; WBC, white blood cell count; LYM%, lymphocyte percentage; IL-6, interleukin-6; CRP, C-reactive protein; PCT, procalcitonin; ESR, erythrocyte sedimentation rate. Between-group comparisons were performed using the Mann–Whitney U test for continuous variables and the Chi-square test or Fisher’s exact test for categorical variables, as appropriate.

**Table 3:** Cox Regression Analysis for Adverse Outcomes and Reinfection.

	Adverse Outcomes						Reinfection					
	Univariate Analysis			Multivariate Analysis			Univariate Analysis			Multivariate Analysis		
Parameter	HR	95% CI	*p*	HR	95% CI	*p*	HR	95% CI	*p*	HR	95% CI	*p*
Opacity score	3.59	1.40–9.22	0.01	3.02	1.17–7.81	0.02	6.06	1.39–26.36	0.02	5.32	1.21–23.46	0.03
Lung volume	0.83	0.44–1.58	0.57				1.21	0.48–3.04	0.69			
Volume of opacities	2.15	1.06–4.36	0.03				2.18	0.85–5.62	0.11			
Percentage of opacities	2.63	1.27–5.45	0.01	2.33	1.12–4.84	0.02	1.92	0.76–4.86	0.17			
Volume of high-attenuation opacities	2.43	1.24–4.77	0.01				3.68	1.43–9.49	0.01	3.81	1.46–9.97	0.01
Percentage of high-attenuation opacities	1.84	0.94–3.59	0.08				3.24	1.26–8.37	0.02	3.39	1.29–8.90	0.01
Mean HU total	2.48	1.28–4.82	0.01	2.20	1.12–4.30	0.02	2.41	0.96–6.07	0.06			
Mean HU of opacity	1.58	0.83–2.99	0.16				2.25	0.89–5.69	0.09			

Data are presented as hazard ratios (HRs) with 95% confidence intervals (CIs) and *p* values derived from Cox proportional-hazards models. Multivariable analyses for adverse outcomes were adjusted for age and COPD, whereas multivariable analyses for reinfection were adjusted for PCT. Abbreviations: CI, confidence interval; COPD, chronic obstructive pulmonary disease; HR, hazard ratio; HU, Hounsfield unit; PCT, procalcitonin. Multivariate analysis for adverse outcomes was adjusted for age and COPD, while the analysis for reinfection was adjusted for PCT.

## Data Availability

The data presented in this study are available on request from the corresponding author. The data are not publicly available due to restrictions concerning patient privacy and ethical compliance, as stipulated by the ethics committee and the data protection policies of our institution.

## Supplementary Materials

The following supporting information can be downloaded at: https://www.mdpi.com/article/10.3390/diagnostics15243156/s1, Table S1: Multivariable Logistic Regression for Adverse Outcomes and Reinfection.
