# Supplementary material for: AI-Based Quantitative HRCT for In-Hospital Adverse Outcomes and Exploratory Assessment of Reinfection in COVID-19
Source: Diagnostics (Basel). 2025 Dec 11;15(24):3156. doi: 10.3390/diagnostics15243156 (PMC12732024; doi:10.3390/diagnostics15243156)
Supplement: Supplementary file 1 [file diagnostics-15-03156-s001.zip › diagnostics-3971108-supplementary.pdf]

Supplementary Table S1. Multivariable Logistic Regression for Adverse Outcomes and Reinfection

	Adverse Outcomes			Reinfection		
Parameter	OR	95 % CI	*P*	OR	95 % CI	*P*
Opacity score	4.07	1.50–11.00	0.01	5.38	1.18–24.62	0.03
Lung volume						
Volume of opacities						
Percentage of opacities	4.11	1.87–9.04	<0.01			
Volume of high-attenuation opacities				3.99	1.40–11.40	0.01
Percentage of high-attenuation opacities				3.47	1.22–9.91	0.02
Mean HU total	4.03	1.91–8.51	<0.01			
Mean HU of opacity						
